# RETRACTION: Effects of Quality Nursing on the Surgical Site Wound Infections in Patients Undergoing Cardiothoracic Surgery: A Meta‐Analysis

**DOI:** 10.1111/iwj.70284

**Published:** 2025-03-03

## Abstract

**RETRACTION:** LvX. , ZhouA. , ChenM. , QiC. , and ZhangQ. , “Effects of Quality Nursing on the Surgical Site Wound Infections in Patients Undergoing Cardiothoracic Surgery: A Meta‐Analysis,” International Wound Journal 21, no. 1 (2024): e14553, 10.1111/iwj.14553.38272809 PMC10789546

The above article, published online on 15 January 2024, in Wiley Online Library (http://onlinelibrary.wiley.com/), has been retracted by agreement between the journal Editor in Chief, Professor Keith Harding; and John Wiley & Sons Ltd. Following an investigation by the publisher, all parties have concluded that this article was accepted solely on the basis of a compromised peer review process. The editors have therefore decided to retract the article. The authors did not respond to our notice regarding the retraction.

**RETRACTION:** X. Lv , A. Zhou , M. Chen , C. Qi , and Q. Zhang , “Effects of Quality Nursing on the Surgical Site Wound Infections in Patients Undergoing Cardiothoracic Surgery: A Meta‐Analysis,” International Wound Journal 21, no. 1 (2024): e14553, 10.1111/iwj.14553.38272809 PMC10789546

The above article, published online on 15 January 2024, in Wiley Online Library (http://onlinelibrary.wiley.com/), has been retracted by agreement between the journal Editor in Chief, Professor Keith Harding; and John Wiley & Sons Ltd. Following an investigation by the publisher, all parties have concluded that this article was accepted solely on the basis of a compromised peer review process. The editors have therefore decided to retract the article. The authors did not respond to our notice regarding the retraction.

